# Fabrication of 3D Printing Scaffold with Porcine Skin Decellularized Bio-Ink for Soft Tissue Engineering

**DOI:** 10.3390/ma13163522

**Published:** 2020-08-10

**Authors:** Su Jeong Lee, Jun Hee Lee, Jisun Park, Wan Doo Kim, Su A Park

**Affiliations:** 1 Medical Device Convergence Center, Konyang University Hospital, 158 Gwanjedong-Ro, Seo-Gu, Daejeon 35365, Korea; sujeong@kyuh.ac.kr; 2 Department of Nature-Inspired Nano Convergence Systems, Nano-Convergence Manufacturing Systems Research Division, Korea Institute of Machinery & Materials (KIMM), 156 Gajeongbuk-Ro, Yuseong-Gu, Daejeon 34103, Korea; meek@kimm.re.kr; 3 Medical Device Development Center, Osong Medical Innovation Foundation, 123 Osongsaengmyung-Ro, Osong-Eub, Heungdeok-Gu, Cheonju-Si, Chungbuk 28160, Korea; owner5306@kbiohealth.kr

**Keywords:** 3D bioprinting, bio-ink, decellularized extracellular matrix, skin dermis

## Abstract

Recently, many research groups have investigated three-dimensional (3D) bioprinting techniques for tissue engineering and regenerative medicine. The bio-ink used in 3D bioprinting is typically a combination of synthetic and natural materials. In this study, we prepared bio-ink containing porcine skin powder (PSP) to determine rheological properties, biocompatibility, and extracellular matrix (ECM) formation in cells in PSP-ink after 3D printing. PSP was extracted without cells by mechanical, enzymatic, and chemical treatments of porcine dermis tissue. Our developed PSP-containing bio-ink showed enhanced printability and biocompatibility. To identify whether the bio-ink was printable, the viscosity of bio-ink and alginate hydrogel was analyzed with different concentration of PSP. As the PSP concentration increased, viscosity also increased. To assess the biocompatibility of the PSP-containing bio-ink, cells mixed with bio-ink printed structures were measured using a live/dead assay and WST-1 assay. Nearly no dead cells were observed in the structure containing 10 mg/mL PSP-ink, indicating that the amounts of PSP-ink used were nontoxic. In conclusion, the proposed skin dermis decellularized bio-ink is a candidate for 3D bioprinting.

## 1. Introduction

In the field of tissue engineering, three-dimensional (3D) bioprinting systems can be used to fabricate designed 3D constructs for use as artificial tissues or organs with specific shapes and features using various materials and cells [1,2,3,4]. Bioprinting allows for the shape of the 3D construct to be maintained, providing a condition similar to the cellular microenvironment so that living cells can maintain their specific activities and properties [5,6]. To successfully manufacture bioprinting constructs, it is important to select a material (bio-ink) that can form a shape capable of mimicking biological feedback [7,8]. An optimized bio-ink should have specific physicochemical properties, such as mechanical, rheological, chemical, and biological characteristics [9].

Hydrogels have numerous attractive properties for use as scaffolds in tissue engineering. They are biocompatible and biodegradable, and possess specific cell binding sites that are suitable for cell attachment, growth, and differentiation. Thus, hydrogels show potential as bio-inks, and many studies have used hydrogels in bioprinting applications. Hydrogel materials, including alginate, collagen, fibrinogen, agarose, chitosan, and silk, are used as bio-inks [10,11,12,13,14]. Alginate is a natural material and similar to glycosaminoglycans, which is found in the extracellular matrix (ECM) of the human body. Particularly, it has been widely used as a bio-ink because of its fast and simple gelation properties and good printability [15,16]. However, although alginate shows good printability at high concentration, there are some limitations to its applicability as a bio-ink, such as its lack of suitable cellular activity. Modified forms of alginate can be used as bio-inks for bioprinting applications [17], and it has been combined with other biomaterials, including collagen, gelatin, agarose, fibrin, thrombin, to prepare composite bio-inks [9,10,18,19].

As a new material of bio-ink, ECM was extracted by a decellularization process to form tissues or organs. The ECM is a necessary ingredient in cells to survive and proliferation. Additionally, there were shown to provides physical and mechanical microenvironment of cells [20,21,22]. Some research groups have been studies 3D bioprinting process using various tissue derived decellularized ECM bio-inks [21,23,24]. Decellularized ECM bio-inks expect to help the proliferation and differentiation of cells that the presence of biomolecules, such as growth factors.

**Scheme 1:**
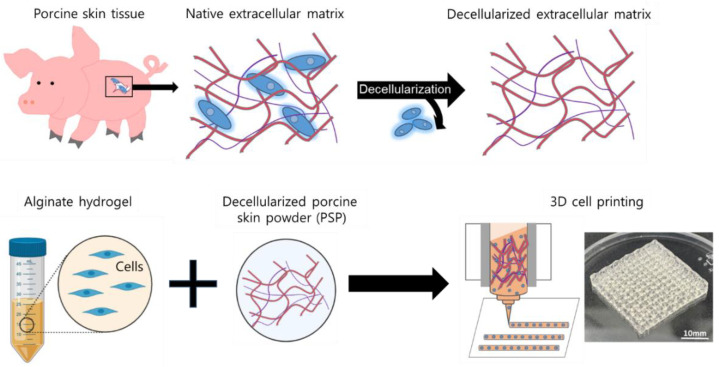
Schematic diagrams of the fabrication process of porcine skin powder (PSP)-inks using decellularization porcine skin tissue.

In this study, bio-ink formulations that are soft tissue-specific were developed by including decellularized porcine skin powder (PSP). This study was conducted to determine whether cells in PSP-ink possessed suitable rheological properties, biocompatibility, and ECM formation after 3D printing (Scheme 1).

## 2. Materials and Methods

### 2.1. Preparation of PSP

Decellularization of the porcine dermis was performed as previous described with some modifications [25]. Full-thickness skin was supplied by the Animal and Plant Quarantine Agency and used with approval from the supplier (Daejeon, Korea). Subcutaneous fat and the epidermis were scratched and removed to isolate the dermal layer. The prepared dermis was stored at −80 °C. The dermis was thawed and chopped for decellularization. The pieces of the dermis were soaked in 0.25% trypsin on a shaker (300 rpm) for 6 h, and then washed with deionized water three times for 15 min each time. The chopped tissue was treated with 70% ethanol for 10 h, treated with 3% H_2_O_2_ for 15 min, and then washed twice for 15 min each time with de ionized water. These samples were treated with 1% Triton X-100 in 0.26% EDTA/0.69% Tris for 6 h, followed by the same treatment for an additional 18 h. The samples were washed twice for 15 min each time with deionized water. The decellularized skin tissues were frozen and lyophilized. To manufacture PSP, the lyophilized skin tissues were ground into a powder with a cryogenic grinder (Freezer/Mill, Spex Sampleprep, Rickmansworth, UK) and filtered through a 100-µm pore mesh.

### 2.2. Decellularization Evaluation of PSP

To identify whether decellularization was complete, native and decellularized tissues were confirmed by diamidino-2-phenylindole (DAPI) fluorescence staining and hematoxylin and eosin (H&E) staining. For nuclear counterstaining, the tissues were fixed and incubated with DAPI solution to counterstain the cell nuclei, and then observed with a fluorescence microscope (ECLIPSE-Ti, Nikon, Tokyo, Japan). For histological evaluation, samples were fixed in 4% formalin, embedded in paraffin, sectioned at 4 μm, stained with H&E, and observed under microscope.

### 2.3. Characterization of Bio-Ink Containing PSP (PSP-Ink)

#### 2.3.1. Fourier-Transform Infrared Spectroscopy (FT-IR)

The chemical structure characterization of PSP was conducted by infrared spectroscopy. The infrared spectra of PSP were measured using a 2000 Fourier-transform infrared spectroscopy (FT-IR) spectrophotometer (PerkinElmer, Waltham, MA, USA). Type I collagen (Gibco, Grand Island, NY, USA) was analyzed as a control. FT-IR analysis was performed at wavelengths of 4000 to 450 cm^−1^.

#### 2.3.2. Rheometer

The mechanical properties of the fabricated PSP-inks were measured with a rheometer (KINEXUS, Malvern Instruments, Malvern, UK). PSP-inks were prepared to 10 and 20 mg/mL PSP in 2% sodium alginate hydrogel (A2033, Sigma-Aldrich, St. Louis, MO, USA), that alginate hydrogel was dissolved in Dulbecco’s modified Eagle’s medium (DMEM, HyClone, Logan, UT, USA) containing 1% (*w*/*v*) CaCl_2_. Rheological measurement was performed at a frequency sweep measurement of 0.1–10 Hz with 0.04 strain. Young’s modulus was calculated from the obtained shear modulus at 1 Hz, as previously described [17].

### 2.4. 3D Bioprinting Characterization of Cell-Laden Construct

#### 2.4.1. Cell Culture and Cell Printing

A mouse embryonic fibroblast cell line (NIH3T3, Korean Cell Line Bank, Seoul, Korea) was cultured in high-glucose DMEM supplemented with 10% fetal bovine serum (Gibco) and 1% antibiotic solution (10,000 U/mL penicillin and 10 mg/mL streptomycin, Gibco). The cultures were maintained in a 37 °C incubator at 5% CO_2_. The cells were passaged at 80% confluence where the plating density was approximately 7.3 × 10^3^ cells/cm^2^. For cell printing, the cells (1 × 10^6^ cells/mL) were mixed with PSP-ink (10 or 20 mg/mL in 2% alginate hydrogel) or 3% alginate hydrogel in the cell medium. The 3D cell laden structures composed of PSP-inks were produced using a custom-made 3D bioprinting system at the Korea Institute of Machinery and Materials (Daedeok-gu, Korea). All bio-inks were printed through a straight ceramic nozzle (500 µm diameter) at an extrusion rate of 220 mm/s. The printed structures were crosslinked by soaking 5% CaCl_2_ in distilled water for 2 min, and washed with phosphate-buffered saline. The 3D structures were cultured in cell culture medium and in a 37 °C incubator at 5% CO_2_.

#### 2.4.2. Cellular Activity of 3D Printed Cells

The cell metabolic activity and viability of the 3D-printed cells were analyzed using a WST-1 cell proliferation assay kit (Takara Bio, Shiga, Japan) at 1, 4, and 7 days. For analysis, we prepared samples with a printed structure of 6 × 10 mm^2^ in size. WST-1 solution and DMEM without fetal bovine serum were mixed and added to individual sample-containing 96-wells. The plate was incubated for 20 min in a 37 °C and 5% CO_2_ incubator. After incubation, 200 µL of supernatant from each well was transferred into a new 96-well plate, and absorbance was measured at 450 nm using a microplate spectrophotometer (Bio-Rad, Hercules, CA, USA).

Encapsulated cells in the bio-inks were evaluated using a Live/Dead Viability/Cytotoxicity Kit (Invitrogen, Carlsbad, CA, USA), according to the manufacturer’s instructions. The cell-laden structures were prepared at 7 days after 3D printing, washed, and stained with 2 mM calcein AM (live cell stain) and 4 mM ethidium homodimer-1 (dead cell stain) solution at 37 °C for 20 min. After incubation, live/dead assay solution was removed, and the samples were washed with phosphate-buffered saline. Live and dead cells were observed by fluorescence microscopy (ECLIPSE-Ti, Nikon, Tokyo, Japan) and images were captured using NIS-Elements Imaging Software 3.2 (Nikon, Tokyo, Japan).

#### 2.4.3. ECM Component Assay of 3D Printing Construct

To evaluate ECM component formation in the 3D cell laden constructs at 7 days after 3D printing, the soluble collagen and elastin contents were examined. Before the ECM component assay, all samples were lyophilized and weighed. Soluble collagen was quantified using the Sircol Soluble Collagen Assay kit (Biocolor Ltd., Belfast, UK) according to the manufacturer’s instructions. Briefly, the sample was added to 1 mL of Sircol Dye Reagent (colorimetric reagent) and agitated for 30 min, followed by centrifugation at 12,000 rpm for 10 min. Sircol Dye was released from the pellet with alkali reagent and spectrophotometric readings were taken at 555 nm on a microplate spectrophotometer (Bio-Rad, Hercules, CA, USA). The recorded values were normalized by a standard curve using a collagen standard of 500 μg/mL supplied with the kit and expressed as a ratio to the dry weight. Elastin content was measured in the lyophilized samples with the Fastin Elastin assay kit (Biocolor Ltd., Carrickfergus, UK).

To extract as alpha-elastin the lyophilized sample was heated at 100 °C for 1 h with 0.25 M oxalic acid. The sample was precipitated with an equal volume of Elastin Precipitating Reagent for 10 min, and soluble alpha-elastin was collected by centrifugation at 10,000 rpm for 10 min. For result quantified, alpha-elastin standard used a recombinant alpha-elastin (12.5, 25, and 50 μg) supplied with the kit. To formation of elastin-dye complex, extracts were reacted with 1 mL of dye reagent for 1.5 h on a mechanical shaker, and centrifuged at 10,000 rpm for 10 min. After the supernatant was removed, the pellet was solubilized with 250 μL of dye-dissociation reagent. Elastin content was measured at a wavelength of 513 nm using a spectrophotometer (Bio-Rad, Hercules, CA, USA), and quantified by extrapolation against the standard curve. Elastin content was expressed as μg/mg dried sample weight.

### 2.5. Statistical Analysis

Data were presented as the mean ± standard deviation (SD), unless otherwise noted. Statistical significance was determined by one-way ANOVA with Tukey’s post hoc test, using GraphPad Prism version 8.4 software (GraphPad Software, San Diego, CA, USA). Values of *p* < 0.05 were regarded as statistically significant. Statistical significance was assigned as * *p* < 0.05, ** *p* < 0.01, or *** *p* < 0.001.

## 3. Results and Discussion

As cell-friendly materials, hydrogels have been widely used in 3D bioprinting to fabricate artificial tissues or organs. For tissue-specific bio-ink materials, researchers have developed decellularized tissue-based bio-inks to mimic the specific natural environments of various tissue types. In this study, PSP-ink composed of extracellular matrix excluding the nucleus was prepared from porcine skin tissue and confirmed to be biocompatible and useful for 3D printing.

**Figure 1:**
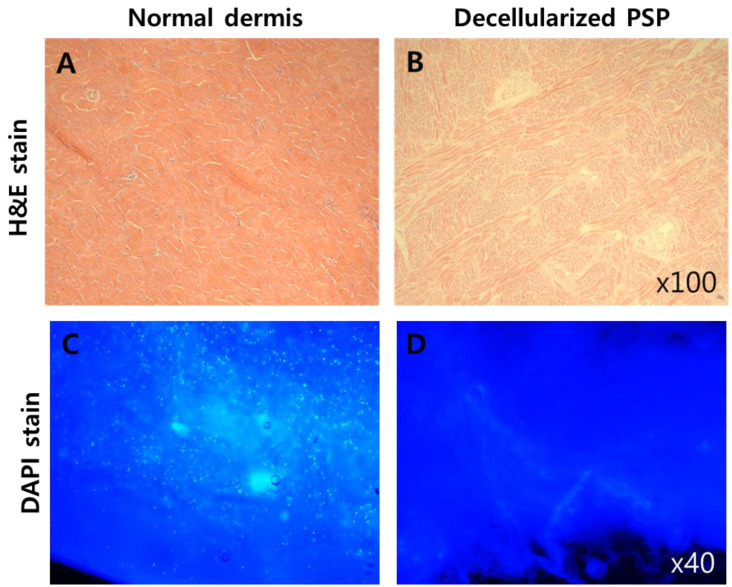
Qualitative analysis of decellularized porcine skin powder (PSP). Hematoxylin and eosin (H&E) (**A**,**B**) and diamidino-2-phenylindole (DAPI) stains (**C**,**D**) show the preserved extracellular matrix (ECM) without cells (nucleus).

To produce 3D printing bio-ink for soft tissue, decellularization was accomplished to remove only cells from the porcine dermis. To evaluate the state of decellularization, DAPI and H&E staining were carried out. As shown in Figure 1, the nuclei of cells in decellularized skin were not stained compared to those in cells from normal skin, whereas the ECM of tissue was maintained.

**Figure 2:**
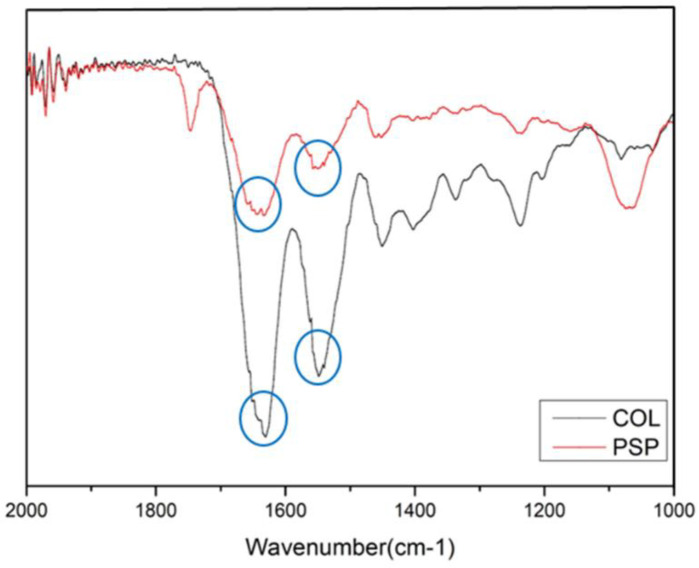
Fourier-transform infrared spectroscopy (FT-IR) spectra of the PSP and collagen type Ⅰ.

To confirm the elemental composition of the PSP compared to collagen, FT-IR experiments were performed: the results are shown in Figure 2. The spectrum of PSP contained peaks at 1651 cm^−1^ (C=O stretching, amide Ⅰ) and 1558–1542 cm^−1^ (N-H deformation, amide Ⅱ). The classical absorption bands of proteins (amide Ⅰ and Ⅱ) are found on the spectra as PSP, which are very close to the absorption bands of type Ⅰ collagen. This result is also similar to the FT-IR spectra of fresh explant (skin dermis) [26].

**Figure 3:**
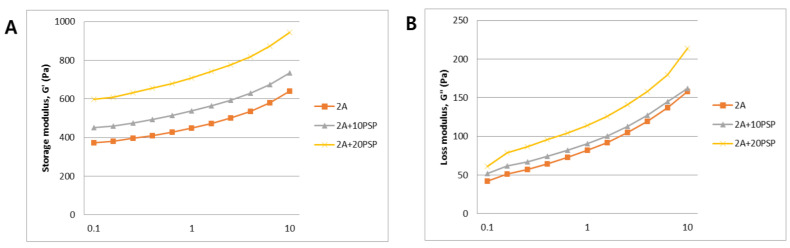
Mechanical properties of PSP-inks. The storage modulus G’ (**A**) and the loss modulus G” (**B**) were plotted logarithmically against frequency (0.1–10 Hz at 24 °C).

Most 3D bioprinting methods using alginate have utilized alginate alone, in a modified form, or blended with other biomaterials as bio-inks. One research group developed a method for producing cell-laden constructs with good mechanical and biological properties [11,16,27]. Notably, increasing the alginate concentration from 2% to 6% (*w*/*v*) reduced cell viability, because of the increased viscosity. Our previous study demonstrated that the viscosity of bio-inks is very important for achieving successful 3D bioprinting and that 3% alginate was optimal for use as a bio-ink [15]. In this study, a minimum concentration of 2% alginate was selected, and viscosity was compared by mixing PSP at two concentrations. As shown in Figure 3, viscosity measured at increasing PSP concentrations of 10–20 mg/mL was significantly increased compared to with 2% alginate only. These results suggest that our PSP-inks enabled fabrication of 3D-printed constructs with suitable viscosity properties.

**Figure 4:**
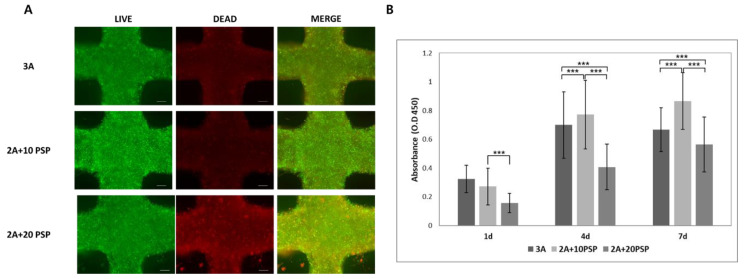
Cellular metabolic activity and viability of the printed cell in PSP-inks. (**A**) Live/Dead staining fluorescence image. Scale bars are 200 μm. (**B**) WST-1 assay results of printed cells in PSP-inks after 7 days of incubation. The statistical significance of differences between the 3% alginate hydrogel (3A) and PSP-inks in 2% alginate (2A+10PSP, 2A_20PSP) groups were assessed using one-way ANOVA with Tukey’s post hoc test. *** *p* < 0.001.

To confirm the biocompatible PSP-ink concentration for cell metabolic activity, we performed cell viability analysis. As shown in Figure 4A, live cell portions were approximately ~75% in the cell-laden construct. The metabolic activity of the printed cells steadily increased during the 7 days in all samples. In particular, cells printed with the 10 mg/mL PSP-ink formulation showed the highest metabolic activity compared to cells printed with other bio-inks. In the 20 mg/mL PSP-ink, cellular activity was even lower than in the 10 mg/mL PSP-ink samples or 2% alginate control ink sample. These results suggest that a high extracellular matrix concentration inhibits cell growth. Therefore, further studies are needed to determine the optimal concentration of extracellular matrix-induced bio-inks.

**Figure 5:**
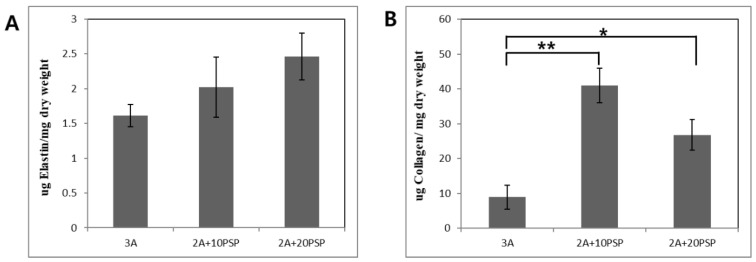
Quantitative analysis of the ECM components. Elastin contents (**A**) and collagen contents (**B**) results of the cells printed structures after 7 days of incubation. The statistical significance of differences between the 3% alginate hydrogel (3A) and PSP-inks in 2% alginate (2A+10PSP, 2A_20PSP) groups were assessed using one-way ANOVA. * *p* < 0.05 and ** *p* < 0.01.

To demonstrate the potential for using PSP-ink in 3D constructs, we cultured 3D constructs of printed cells using PSP-ink. After 7 days of culture, we analyzed the main extracellular matrix components of the skin, collagen and elastin (Figure 5). Quantification analysis showed that the proportion of elastin was well-retained, particularly PSP was significantly increased when compared with 2% alginate control ink sample. In contrast, cells printed with the 10 mg/mL PSP-ink formulation showed the highest collagen contents compared to 20 mg/mL PSP-ink. The results are similar to those of cell metabolic activity analysis (WST-1 and live/dead staining). The secretion of ECM proteins is important factor demonstrating the potential of bio-ink to induce cellular responses, as the cells are structurally and biochemically controlled by surrounding ECM components [28]. Our results suggested the potential as 3D printing material of PSP-inks by evaluating cell metabolic activity, as well as collagen production in cell-laden 3D construct used 10 mg/mL PSP-ink.

## 4. Conclusions

In summary, we fabricated and characterized bio-inks based on the biocompatible material PSP using a decellularization process. A suitable bio-ink capable of supporting the function of printed cells was developed. Studies are needed to confirm the cellular activity and mechanical properties in bio-ink. Our PSP-ink was fabricated as a 3D construct, and was confirmed to have suitable biocompatibility and to produce ECM components. We also confirmed that a sufficient amount of PSP is not required for soft tissue engineering application research, but an appropriate concentration is sufficient.

## References

[1] Murphy S.V., Atala A. (2014). 3D bioprinting of tissues and organs. Nat. Biotechnol..

[2] Billiet T., Vandenhaute M., Schelfhout J., Van Vlierberghe S., Dubruel P. (2012). A review of trends and limitations in hydrogel-rapid prototyping for tissue engineering. Biomaterials.

[3] Derby B. (2012). Printing and prototyping of tissues and scaffolds. Science.

[4] Ozbolat I.T., Hospodiuk M. (2016). Current advances and future perspectives in extrusion-based bioprinting. Biomaterials.

[5] Loo Y., Hauser C.A. (2015). Bioprinting synthetic self-assembling peptide hydrogels for biomedical applications. Biomed. Mater..

[6] Yang J., Zhang Y.S., Yue K., Khademhosseini A. (2017). Cell-laden hydrogels for osteochondral and cartilage tissue engineering. Acta Biomater..

[7] Chia H.N., Wu B.M. (2015). Recent advances in 3D printing of biomaterials. J. Biol. Eng..

[8] Park J.H., Jang J., Lee J.S., Cho D.W. (2017). Three-dimensional printing of tissue/organ analogues containing living cells. Ann. Biomed. Eng..

[9] Lee H.J., Kim Y.B., Ahn S.H., Lee J.S., Jang C.H., Yoon H., Chun W., Kim G.H. (2015). A new approach for fabricating collagen/ecm-based bioinks using preosteoblasts and human adipose stem cells. Adv. Healthc. Mater..

[10] Gungor-Ozkerim P.S., Inci I., Zhang Y.S., Khademhosseini A., Dokmeci M.R. (2018). Bioinks for 3D bioprinting: An overview. Biomater. Sci..

[11] Gao Q., He Y., Fu J.Z., Liu A., Ma L. (2015). Coaxial nozzle-assisted 3D bioprinting with built-in microchannels for nutrients delivery. Biomaterials.

[12] Moon S., Hasan S.K., Song Y.S., Xu F., Keles H.O., Manzur F., Mikkilineni S., Hong J.W., Nagatomi J., Haeggstrom E. (2010). Layer by layer three-dimensional tissue epitaxy by cell-laden hydrogel droplets. Tissue Eng. Part C Methods.

[13] Cui X., Boland T. (2009). Human microvasculature fabrication using thermal inkjet printing technology. Biomaterials.

[14] Das S., Pati F., Choi Y.J., Rijal G., Shim J.H., Kim S.W., Ray A.R., Cho D.W., Ghosh S. (2015). Bioprintable, cell-laden silk fibroin-gelatin hydrogel supporting multilineage differentiation of stem cells for fabrication of three-dimensional tissue constructs. Acta Biomater..

[15] Park J., Lee S.J., Chung S., Lee J.H., Kim W.D., Lee J.Y., Park S.A. (2017). Cell-laden 3D bioprinting hydrogel matrix depending on different compositions for soft tissue engineering: Characterization and evaluation. Mater. Sci. Eng. C Mater. Biol. Appl..

[16] Zhang Y., Yu Y., Chen H., Ozbolat I.T. (2013). Characterization of printable cellular micro-fluidic channels for tissue engineering. Biofabrication.

[17] Park J., Lee S.J., Lee H., Park S.A., Lee J.Y. (2018). Three dimensional cell printing with sulfated alginate for improved bone morphogenetic protein-2 delivery and osteogenesis in bone tissue engineering. Carbohydr. Polym..

[18] Nakamura M., Iwanaga S., Henmi C., Arai K., Nishiyama Y. (2010). Biomatrices and biomaterials for future developments of bioprinting and biofabrication. Biofabrication.

[19] Zhang T., Yan K.C., Ouyang L., Sun W. (2013). Mechanical characterization of bioprinted in vitro soft tissue models. Biofabrication.

[20] Choudhury D., Tun H.W., Wang T., Naing M.W. (2018). Organ-derived decellularized extracellular matrix: A game changer for bioink manufacturing?. Trends Biotechnol..

[21] Jang J., Park H.J., Kim S.W., Kim H., Park J.Y., Na S.J., Kim H.J., Park M.N., Choi S.H., Park S.H. (2017). 3D printed complex tissue construct using stem cell-laden decellularized extracellular matrix bioinks for cardiac repair. Biomaterials.

[22] Pati F., Ha D.H., Jang J., Han H.H., Rhie J.W., Cho D.W. (2015). Biomimetic 3D tissue printing for soft tissue regeneration. Biomaterials.

[23] Garreta E., Oria R., Tarantino C., Pla-Roca M., Prado P., Fernández-Avilés F., Campistol J.M., Samitier J., Montserrat N. (2017). Tissue engineering by decellularization and 3D bioprinting. Materialstoday.

[24] An J., Teoh J.E.M., Suntornnond R., Chua C.K. (2015). Design and 3D printing of scaffolds and tissues. Engineering.

[25] Wolf M.T., Daly K.A., Brennan-Pierce E.P., Johnson S.A., Carruthers C.A., D’Amore A., Nagarkar S.P., Velankar S.S., Badylak S.F. (2012). A hydrogel derived from decellularized dermal extracellular matrix. Biomaterials.

[26] Tang R., Samouillan V., Dandurand J., Lacabanne C., Nadal-Wollbold F., Casas C., Schmitt A.-M. (2017). Thermal and vibrational characterization of human skin. J. Therm. Anal. Calorim..

[27] Yu Y., Zhang Y., Martin J.A., Ozbolat I.T. (2013). Evaluation of cell viability and functionality in vessel-like bioprintable cell-laden tubular channels. J. Biomech. Eng..

[28] Streuli C. (1999). Extracellular matrix remodelling and cellular differentiation. Curr. Opin. Cell Biol..

